# Microbiome–Metabolome Axis in BALF Reveals Novel Diagnostic Biomarkers for Congenital Heart Disease-Associated Pulmonary Arterial Hypertension

**DOI:** 10.3390/jcdd13010032

**Published:** 2026-01-06

**Authors:** Xiaoyu Zhang, Liming Cheng, Yuan Zhou, Jiahui Xie, Wenting Gui, Jiaxiang Chen, Zidan Zhang, Kai Liu, Runwei Ma

**Affiliations:** 1 Department of Cardiac Surgery, Fuwai Yunnan Hospital, Chinese Academy of Medical Sciences/Affiliated Cardiovascular Hospital of Kunming Medical University, Kunming 650032, China; zhangxiaoyu@kmmu.edu.cn (X.Z.);; 2 Department of Anesthesiology, Kunming Children’s Hospital, Kunming 650034, China; medcheng@163.com; 3 Department of Comprehensive Pediatrics, Kunming Children’s Hospital, Kunming 650034, China

**Keywords:** congenital heart disease, pulmonary arterial hypertension, microbiome, metabolomics, biomarker, mediation analysis

## Abstract

**Background:** Early identification of irreversible pulmonary vascular remodeling in congenital heart disease-associated pulmonary arterial hypertension (C-PAH) is critical for optimizing surgical timing. Current noninvasive diagnostic methods are inadequate, and the lung microbiome and metabolome may provide novel insights into disease progression. **Methods:** We analyzed bronchoalveolar lavage fluid (BALF) from 47 children, including those with C-PAH (n = 15), CHD without PAH (C-NPAH, n = 16), and healthy controls (n = 16), using 16S rRNA gene sequencing and untargeted metabolomics. Differential microbial taxa and metabolites were identified, and their interactions with clinical indicators were assessed via Random Forest (RF) and Mediation Analysis. **Results:** C-PAH patients exhibited airway microbial dysbiosis, characterized by an elevated Firmicutes/Bacteroidetes (F/B) ratio and increased abundance of *g_Lactobacillus*. Metabolomic profiling revealed 88 differential metabolites between C-PAH and controls, and 3 between C-PAH and C-NPAH. N1-methylnicotinamide (MNAM) and 2-piperidone emerged as potential biomarkers. Mediation analysis showed that *g_Eikenella* influenced PAH indirectly through 2-piperidone (β = −0.376, *p* = 0.026), indicating a microbe–metabolite–host interaction. **Conclusions:** Integrative microbiome–metabolome profiling of BALF reveals potential biomarkers for C-PAH. These findings provide exploratory evidence that microbial and metabolic biomarkers, particularly 2-piperidone and MNAM, hold potential for the early, noninvasive identification of irreversible pulmonary vascular remodeling, but require further validation in independent cohorts.

## 1. Introduction

Pulmonary arterial hypertension (PAH) is the most extensively studied subtype of pulmonary hypertension, characterized by pathological changes such as “pre-capillary” pulmonary vascular remodeling [1]. Left-to-right shunt congenital heart disease (LTRS-CHD) is the most important factor contributing to PAH [2]. C-PAH is also emerging as a global challenge [3]. Evidence shows that it remains the predominant type of PAH in the Chinese population [4], but its impact spans international borders, and the disease is a critical public health concern in many countries [5]. According to the pressure ratio of pulmonary/systemic blood flow (Qp/Qs), C-PAH is divided into dynamic and resistance types. Pulmonary vascular lesions in patients with dynamic PAH (Qp/Qs > 1.5) can be reversed, and early defect repair is the key to treatment. Another group of patients with resistance-type PAH develop an irreversible Eisenmenger syndrome stage of pulmonary vascular disease (contraindication for surgical repair), where PAH-targeted drugs can only delay the progression of the disease and cannot reverse pulmonary vascular remodeling. Therefore, effective preoperative identification of reversible and irreversible pulmonary vascular remodeling is crucial for determining the operability of C-PAH, selecting treatment strategies, and accurate prognosis. Unfortunately, there is a current lack of simple and effective noninvasive diagnostic methods and criteria [6,7], and the pathological mechanisms of pulmonary vascular remodeling are still under study.

Traditionally, it is believed that the progression of PAH caused by LTRS-CHD is related to the size of the shunt. However, interestingly, in actual clinical practice, a small number of patients, despite having large LTRSs, maintain surgical indications after undergoing right heart catheterization, and the effect of surgical is beneficial, with rapid postoperative recovery. This phenomenon suggests that the size of the shunt cannot accurately evaluate the condition of PAH [8], and there may be some undiscovered protective mechanism that delays the pathological changes in pulmonary vascular remodeling in such patients.

Studying microbiota and clinical cardiovascular disease is an emerging research field involving multiple mechanisms, including direct microbe–host interactions, immune regulation, and inflammatory responses [9,10]. Although there have been numerous studies in this field in recent years and many academic breakthroughs have been made, related research has focused more on the gut microbiota [11,12,13,14,15], and there are not many reports on the pathogenesis and protective mechanisms of respiratory microbiota and PAH related to congenital heart disease. LTRS-CHD alters the pulmonary microenvironment, so the effects of this are worth exploring. Given that LTRS-CHD alters the pulmonary microenvironment, and the pathogenesis and protective mechanisms of the respiratory microbiota in C-PAH remain unclear, this study aims to reveal the role of pulmonary microecological imbalance in the occurrence and development of C-PAH by comparing the composition of the lower airway microbiota and metabolic profile characteristics among C-PAH patients, C-NPAH, and healthy controls. It also provides experimental evidence for the exploration of early diagnostic biomarkers and potential pathogenic mechanisms of the disease.

## 2. Materials and Methods

### 2.1. Patient Cohort

A total of 47 participants under the age of 14 participated in this study, including 15 patients diagnosed with left-to-right shunt congenital heart disease complicated with PAH (C-PAH), 16 patients with left-to-right shunt congenital heart disease with non-pulmonary arterial pressure (C-NPAH), and 16 children as a control group (CG). Table 1 lists detailed baseline characteristics and inclusion/exclusion criteria. C-PAH is defined as pulmonary artery systolic pressure (PASP) > 35 mmHg. Based on the Bernoulli equation [16], we estimate PASP using transthoracic ultrasound, calculated as follows: (PASP = 4 × [tricuspid regurgitation jet velocity] 2 + estimated right atrial pressure). The Yunnan Fuwai Cardiovascular Hospital Ethics Committee reviewed and approved study participants. The participants’ legal guardians/close relatives provided written informed consent to participate in this study.

### 2.2. BALF Collection

Due to the susceptibility of the lung microbiota to various factors, all bronchoalveolar lavage fluid (BALF) collections in this study followed a standardized protocol. The procedure was uniformly performed by 2 experienced pediatric respiratory endoscopists from two hospitals, with specific operational details as follows:

Preoperative Preparation: All children were fasted for 6 h and deprived of fluids for 4 h prior to the procedure. Intravenous combined anesthesia was administered (induction with propofol 2~4 mg/kg, maintenance with remifentanil 0.1~0.2 μg/(kg·min)), and vital signs were monitored throughout the process. An Olympus BF-XP290 bronchoscope (Tokyo, Japan, Olympus) (pediatric-specific, distal outer diameter 3.1 mm) was used. Prior to the procedure, the bronchoscope was sterilized with ethylene oxide, and the biopsy channel was repeatedly flushed 3 times with sterile normal saline.

Lavage Parameters: Sterile normal saline at 37 °C was used as the lavage fluid, with a total injection volume of 1 mL/kg (single injection volume ≤ 5 mL) administered in 2 divided doses. After each injection, gentle aspiration was performed through the biopsy channel to mix the fluid, and the lavage fluid was recovered using low negative pressure suction (negative pressure value: −80~−100 mmHg).

Recovery Volume Control: The actual recovery volume of each sample was fully recorded. The average recovery volume was 65% ± 8% of the injection volume (range: 58%~78%), and the recovery volume of all samples was ≥50%. For metabolite analysis, concentration data were standardized and corrected based on the recovery volume to reduce the impact of dilution effects.

Contamination Prevention and Control: The transnasal insertion route was adopted for sampling to avoid contact between the bronchoscope and oral/pharyngeal mucosa. Each patient was provided with independent lavage fluid, suction tubes, and collection containers. After sampling, 2 mL of BALF was transferred to a sterile test tube, rapidly frozen in liquid nitrogen, and then stored in a −80 °C refrigerator for subsequent processing.

### 2.3. 16S rRNA Sequencing and Data Processing

Bacterial genomic DNA was extracted from BALF samples using the CTAB/SDS method. After genomic DNA extraction, 1% agarose gel electrophoresis was used to assess the extracted genomic DNA. According to the designated sequencing region, specific primers with barcode or fusion primers with misplaced bases were synthesized, before purifying them with an Agencourt AMPure XP nucleic acid purification kit (Beckman Coulter Brea, Brea, CA, USA). 16S rRNA high-throughput sequencing was performed using the 16S metadata system (genetic). Following the manufacturer’s instructions, a first-step PCR was performed on approximately 3ng of total genomic DNA to amplify the 16S rRNA gene (V3-V4). The purified PCR products were processed for the second step of PCR to construct sequencing libraries. 16S rRNA was sequenced on the Illumina Miseq (San Diego, CA, USA) platform to obtain paired-end reads. The resulting data are stored in Fastq format. Fastqc software (version 2.17.1.14) was used to control raw sequencing data quality and remove low-quality reads and splice sequences. The high-quality sequencing data were subjected to operational taxonomic unit (OTU) clustering analysis to identify different microbial species. The platform and software used were Qiime (version 1.8.0 http://qiime.org/; accessed on 1 October 2025) and VSEARCH (version 2.7.1 https://github.com/torognes/vsearch; accessed on 1 October 2025). The UPARSE method was selected to generate OTUs from clean tags. Based on the OTU clustering results, we used Mothur (https://mothur.org; accessed on 1 October 2025) to analyze rarefaction, whereas R language tools were used to generate dilution curves.

### 2.4. Analysis of Microbial Diversity and Differences Between Groups

Based on genus level abundance, the Shannon index, richness index, and Pielou index uniformity between each group of samples were calculated using the R package vegan (v2.6-4), and the differences between the groups were compared. Based on Bray–Curtis distance, the distance between bacterial communities in each group was calculated. NMDS was performed using R package vegan, and similarity analysis (ANOSIM) was used to analyze the differences between groups. Subsequently, the R package indicspecies was used to analyze the indicator values of each group of samples, and the representative genera in each group were determined according to the FDR < 0.05 standard. At the same time, LEfSe was performed using the OmicStudio tools (https://www.omicstudio.cn/tool/; accessed on 1 October 2025) for each group, and LDA > 3 was used to obtain the different representative genera.

### 2.5. Analysis of Metabolomic Differences Between Groups

For metabolomic analysis, BALF samples were retrieved from liquid nitrogen and thawed on ice. The metabolites were extracted using methanol and L-2-chlorophenylalanine. The instrumental platform used for the LC-MS analysis was the UHPLC-Q Exactive HF-X system. Chromatographic conditions: the chromatographic column was the Acquity UPLC HSS T3 (100 mm × 2.1 mm I.D, 1.8 µm; Waters, Milford, MA, USA); mobile phase A was water (containing 0.1% formic acid), mobile phase B was acetonitrile (containing 0.1% formic acid), mobile phase C was water (containing 6.5 mm NH_4_HCO_3_), and mobile phase D was 95% methanol–water (containing 6.5 mm NH_4_HCO_3_); positive mode mobile phases were A and B, negative mode mobile phases were C and D; the injection volume was 2 μL, and the column temperature was 40 °C. The gradient elution process for the analytes is shown in Table S23. Quality control (QC) samples were prepared by mixing the extraction solutions of all samples in equal volumes. The volume of each QC sample was the same as that of the analytical samples. The QC samples are processed and analyzed in the same way as the analytical samples. In instrumental analysis, a QC sample is inserted after every 5–15 analytical samples to investigate the stability of the whole detection process. The raw data were imported into the ProgenesisQI (version 2.4) metabolomics processing software (WatersCorporation, Milford, MA, USA) for baseline filtering, peak identification, integration, retention time correction, peak alignment, etc. Finally, a data matrix containing information such as retention time, mass charge ratio, and peak intensity was obtained. Then, the software was used to identify the characteristic peaks, match the MS and ms/ms mass spectrometry information with the metabolic database, set the MS mass error to less than 10 ppm, and identify metabolites according to the secondary mass spectrometry matching score (Table S24). The main databases used were http://www.hmdb.ca/ (accessed on 1 October 2025), https://metlin.scripps.edu/ (accessed on 1 October 2025), GNPS, and MoNA. The main equipment and reagents for metabolite detection are listed in the Supplementary Material (Table S25). Bioinformatic analysis was performed using the OmicStudio tools. The vegan package (version 2.5.4) was used to calculate the *p* and R values, the stats package (version 3.5.0) was used for PCA, and the ggplot2 package (ggplot2: 3.3.3) was used to draw the ellipse diagram. PLS-DA analysis (metax: 2.0.0, ggplot2: 3.3.3, OmicStudioClassic: 1.56.0, OmicStudioKits: 3.45.1) was carried out using R version 4.1.3. T-tests combined with multivariate analysis of PLS-DA were used to screen out the differential metabolites between groups (while meeting VIP > 1, *p* < 0.05). Then, an RF classifier was constructed based on the differential metabolites and the reliability of the classification of differential metabolites between groups was verified through 1000 cycles (RandomForest Package 4.7–1.1 parameters: x = X, y = Group, ntree = 1000, mtry = floor(sqrt(ncol(X))), nodesize = 1, importance = TRUE).

### 2.6. Mediation Analysis of Metabolome Microbiome Clinical Phenotype

Traceability analysis of differential metabolites was performed using the MetOrigin 2.0 tool at https://metorigin.met-bioinformatics.cn/home/ (accessed on 1 October 2025). Spearman’s correlation analysis was used to analyze the correlations of differential metabolites between groups and indicator microorganisms. Metorigin 2.0 uses metabolites as mediating variables. First, Spearman’s correlation analysis was performed on the microbiome data, metabolome data, and phenotypic parameters. Sankey network analysis was then used to identify and visualize the metabolites associated with microbial and phenotypic parameters. Finally, we conducted mediation analysis using the MetOrigin 2.0 platform’s mediation analysis module (causal steps approach).

### 2.7. Statistical Analysis

Statistical analyses were performed using SPSS software (version 27.0; IBM Corp., Armonk, NY, USA). Baseline characteristics were summarized as mean ± standard deviation (SD) for continuous variables with an approximately normal distribution, median (interquartile range, IQR) for non-normally distributed continuous variables, and number (percentage) for categorical variables. Normality of continuous variables was assessed prior to group comparisons. Variables with an approximately normal distribution were compared using Student’s *t*-tests, whereas variables that did not meet the normality assumption were compared using the Mann–Whitney U test. For metabolomic analyses, differential metabolites between groups were identified and visualized using volcano plots generated with GraphPad Prism (version 10.0; GraphPad Software, LLC, San Diego, CA, USA). Metabolites were considered significantly different if they met the following criteria: *p* < 0.05, variable importance in projection (VIP) > 1, and a significant fold change (log2FC > 0 or log2FC < 0). In analyses involving multiple metabolite comparisons, FDR correction was applied to control for multiple testing. For hypothesis-driven analyses with a limited number of predefined comparisons, no multiple testing correction was applied. All statistical tests were two-sided, and a *p* value < 0.05 was considered statistically significant.

## 3. Results

### 3.1. Participant Characteristics

Based on the inclusion, a total of 47 BALF specimens were obtained, 15 from patients with C-PAH, 16 from patients with C-NPAH, and 16 from CG individuals. All participants with C-PAH met the clinical definition of PAH, characterized by a PASP of more than 35 mmHg and a PVR (Pulmonary Vascular Resistance) of more than 3 wood units, confirmed by transthoracic echocardiography and right heart catheterization, respectively. Table 2 shows significant differences in age and MPAD between the C-PAH and C-NPAH patients.

### 3.2. Data Quality Control

The dilution curve showed rich diversity in the 16S sequencing data from the 47 BALF samples. The flattened data comprehensively captured the diversity of samples (Figure S1A,B). After flattening, the 16S sequencing data included microbial data from 14 phyla (Figure S1C) and microbial community data from 75 genera (Figure S1D). Proteobacteria comprised the highest proportion of bacteria, followed by Firmicutes.

### 3.3. Relative Abundance Analysis

**Figure 1:**
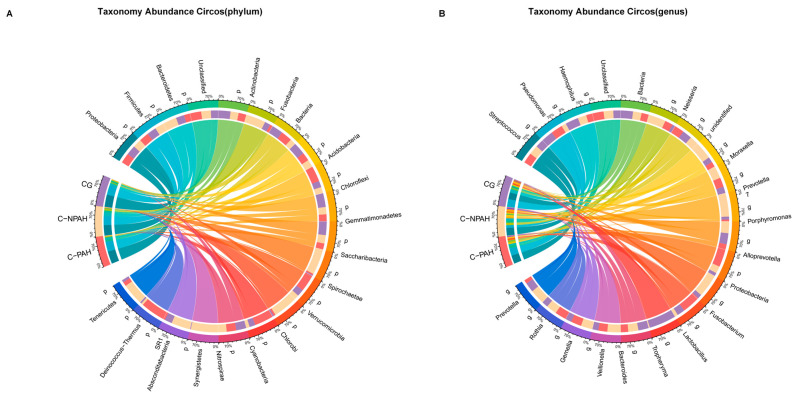
Circos plots of relative abundance. (**A**) The phylum interactions of each group of samples with microorganisms are shown separately. The sectors with different colors in the figure represent different phyla, and the thickness of the lines indicates the interaction strength between samples and species. (**B**) The genus-level interactions of each group of samples with microorganisms are shown separately. Different colored sectors in the figure represent different genus, and the thickness of the line indicates the interaction strength between the sample and the species.

At the phylum level, we selected the top 20 microorganisms in terms of relative abundance for analysis, and the results showed that p_Proteobacteria (43.98) had the highest abundance in the three groups, followed by p_Firmicutes (35.18) (Figure 1A, Table S1). The F/B ratios in the three groups were 3.97 (CG), 2.32 (C-NPAH), and 6.09 (C-PAH), respectively. At the genus level, g_Pseudomonas (21.90) accounted for a high proportion in the CG, while g_Streptococcus was most abundant in the C-PAH (24.34) and C-NPAH groups (22.07). The relative abundance of g_Fusobacterium also significantly differed among the three groups (C-PAH = 0.14, C-NPAH = 3.66, CG = 0.67) (Figure 1B, Table S2).

### 3.4. Microbial Phylum Level Diversity Analysis

We used LEfSe to analyze the bacterial communities in BALF samples and identified potential differential groups. The branching diagram shows that f_Corynebacteriaceae, f_Bacteroidales, f_S24_7_group, and f_Lactobacillaceae are the main taxonomic groups and may be involved in the pathological process of C-PAH. At the genus level, the species are g_Propionibacterium, g_Lactobacillus, g_Castellaniella, and g_Alicycliphilus (Figure S2A, Table S3). In contrast, C-NPAH patients exhibited more characteristic bacteria, including f_Actinomycetaceae, f_Porphyromonadaceae, f_P5D1_392, f_Fusobacteriacea, f_Burkholderiacea, f_Neisseriacea, and f_Campylobacteraceae. Similarly, C-NPAH patients have more characteristic bacteria, such as g_Actinomyces, g_Porphyromonas, g_Catonella, g_Fusobacterium, g_Lautropia, g_Campylobacter, and g_Treponema_2 (Figure S2B, Table S3). Overall, these observations indicate that the composition of the lung microbiota has undergone significant changes between the C-PAH and C-NPAH groups, leading to an ecological imbalance in the lung microenvironment.

### 3.5. Genus-Level Diversity Analysis

**Figure 2:**
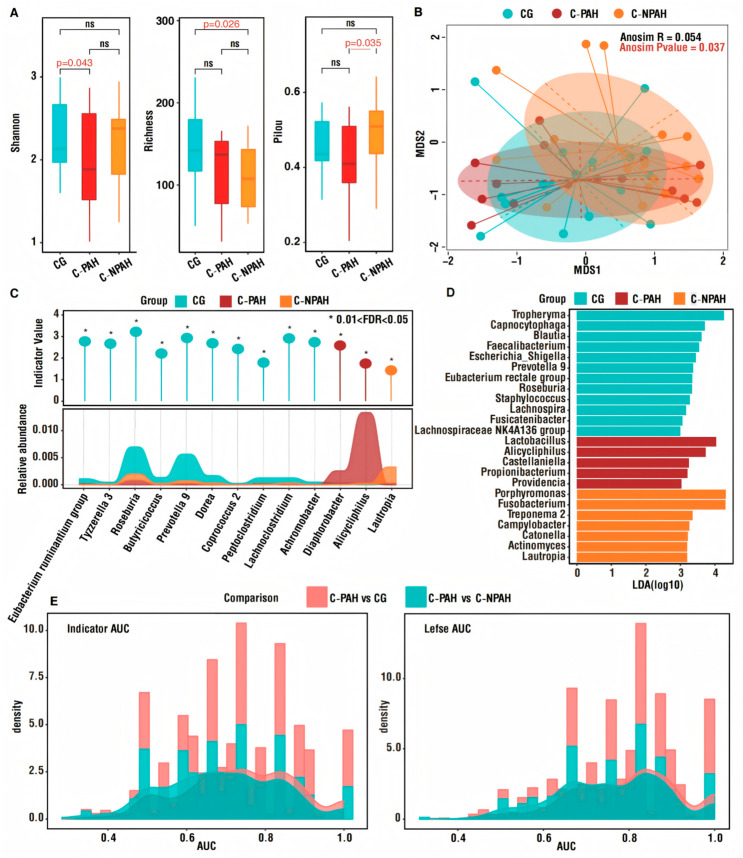
Diversity at the genus level among groups, analysis of differences, and validation of AUC. (**A**) Comparison of Shannon, richness, and Pielou indices among the 3 groups. (**B**) β-diversity analysis among the 3 groups. (**C**) The indicator analysis showed that CG had ten indicators, while the C-PAH group was associated with Diaphorobacter and Alicycliphilus and the C-NPAH group was associated Lautropia (0.01 < FDR < 0.05). (**D**) The results of the LEfSe analysis indicate that there are significant differences in species abundance among the 3 groups. (**E**) An RF classifier was constructed to verify the AUC distribution results after 1000 iterations, suggesting that both have high reliability. AUC = 0.5–0.7, low accuracy; 0.7–0.8, medium accuracy; AUC > 0.8, high accuracy. *p* < 0.05 was considered statistically significant.

Based on the genus-level microbial community analysis, we calculated and compared the Shannon index, richness index, and Pielou index for each group of samples (Figure 2A, Table S4). As shown in Figure 2A, the Shannon index of the CG was significantly higher than that of the C-PAH group, while there was no significant difference between the other two groups. The CG was significantly higher than the C-NPAH group in terms of the Richness index. The Pielou index of the C-NPAH group is significantly higher than that in the C-PAH group, indicating that C-NPAH patients have a higher level of bacterial uniformity (Figure 2A). Based on NMDS and ANOSIM analysis, β diversity analysis was conducted, and the results showed differences among the three groups: ANOSIM R = 0.054, ANOSIM *p* = 0.037 (Figure 2B). Subsequently, based on indicator analysis (0.01 < FDR < 0.05), we found multiple indicators in the CG sample. At the same time, the C-PAH and C-NPAH groups only had two and one indicators, respectively, namely, g_Diaphoribacter and g_Alicyclinophilus, and *g_Lautrobia* (Figure 2C, Table S5). Meanwhile, we conducted LEfSe analysis on the genera of the three sample groups. We found that LEfSe analysis produced more labeled bacteria than indicator value analysis (Figure 2D and Figure S2A,B, Tables S3 and S6). Next, an RF classifier was generated using indicators and LEfSe markers. The AUC distribution was validated 1000 times, and the density curve results show that the indicator and LEfSe have high reliability, with AUC values greater than 0.7 and 0.8, respectively (Figure 2E).

### 3.6. Differential Metabolite Analysis

**Figure 3:**
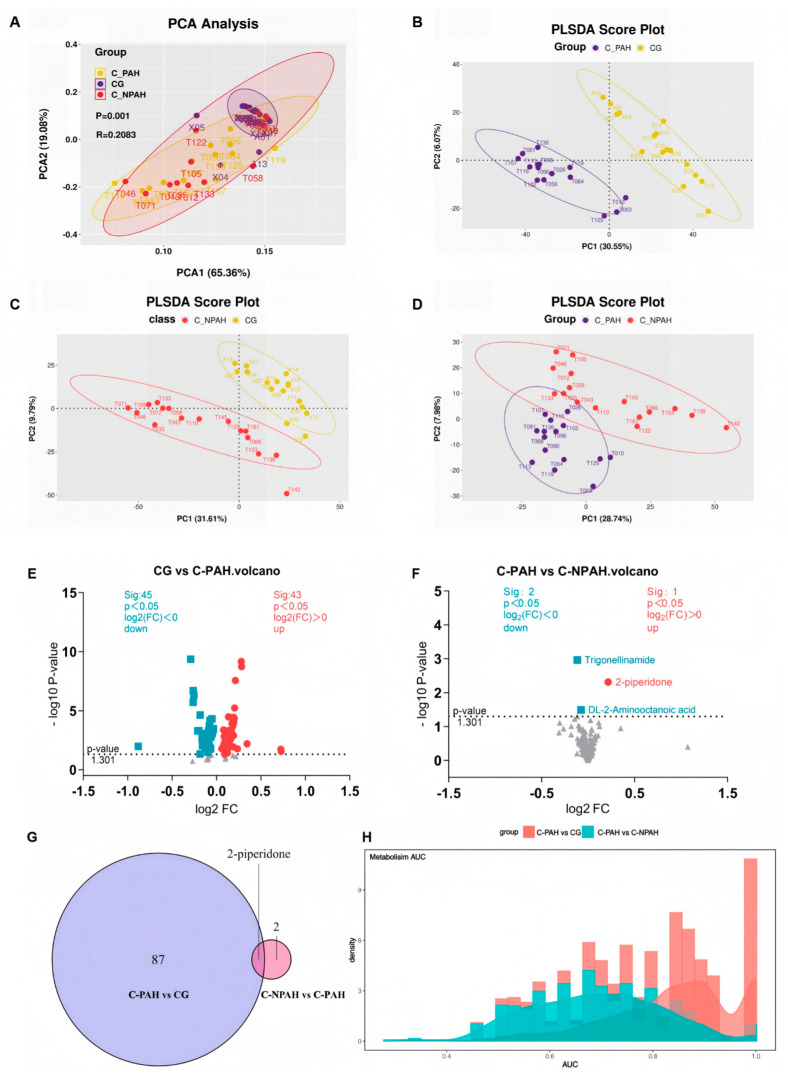
Differential analysis of 3 metabolic groups and RF validation AUC. (**A**) PCA for differential metabolites (*p* = 0.001). PCA1 explained 65.36% of the variance, whereas pca2 explained 19.08%. (**B**–**D**) The PLS-DA calculation results demonstrate the differences and similarities between pairwise samples. (**E**) Volcano plot of differential metabolites between the CG and C-PAH group (88 differential metabolites). (**F**) Volcano plot of differential metabolites between the C-PAH and C-NPAH groups (3 differential metabolites). (**G**) After pairwise comparison, the intersection of differential metabolites was obtained to obtain a common differential metabolite, 2-piperidone. (**H**) An RF classifier was constructed based on 88 and 3 differential metabolites, and the reliability of these differential markers was verified. AUC = 0.5–0.7, low accuracy; 0.7–0.8, medium accuracy; AUC > 0.8, high accuracy. *p* < 0.05 was considered statistically significant.

Principal component analysis (PCA) of the three metabolic datasets revealed significant differences (*p* = 0.001) between the C-PAH, C-NPAH, and control groups. PCA1 and PCA2 together explained 84.44% of the variance (65.36% + 19.08%), indicating that these two principal components can well represent the variation in the original data (Figure 3A, Table S7). PLS-DA analysis was performed on the C-PAH and C-NPAH groups, the CG and C-PAH group, and the CG and C-NPAH group. The score plots showed differences in the analyzed features between the CG and C-NPAH group and between the CG and C-PAH group (Figure 3B,C). Permutation tests were performed on the above results; the Q^2^ intercepts were −0.45 and −0.45, respectively (Figure S3A,B, Table S8), thereby ruling out overfitting. Although there is some similarity between the C-PAH and C-NPAH groups (Figure 3D), the original R^2^ is 0.70 (Figure S3C, Table S8). The original model’s R^2^ value should usually be higher than 0.5; the closer it is to 1, the better the model’s fit. This indicates that the model has strong explanatory power for the data and a certain degree of discriminative ability.

Metabolite difference analysis identified 88 metabolites that differed between the CG and C-PAH group (Figure 3E, Table S9). In contrast, only three differential metabolites were identified between the C-PAH and C-NPAH groups (Figure 3F, Table S10). Furthermore, we took the intersection of two sets of differentially expressed metabolites and obtained a common differentially expressed metabolite, 2-piperidone (Figure 3G, Table S11). The RF classifier and validation results indicate that the differential metabolites between the C-PAH group and CG have better classification reliability than those between the C-PAH and C-NPAH groups. It is worth noting that although there are only three differential metabolites between the C-PAH and C-NPAH groups, the final validation AUC peaks of these three differential metabolites are all above 0.7, indicating the reliability of the results (Figure 3H).

### 3.7. Traceability Analysis of Differential Metabolites

**Figure 4:**
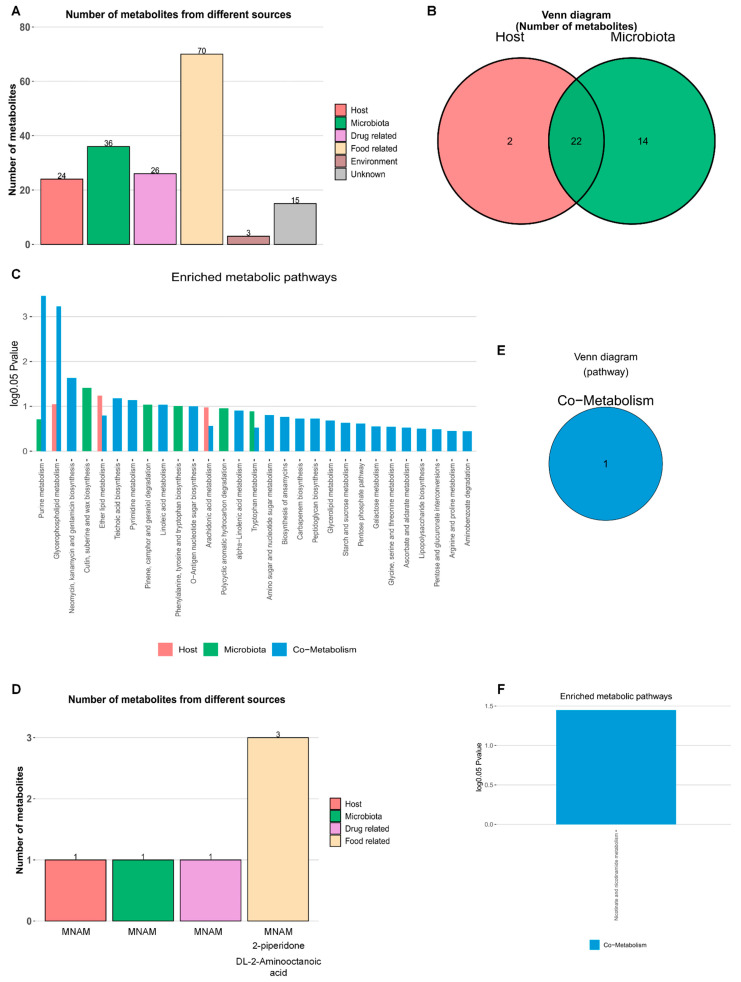
Metabolite traceability analysis and Metabolic Pathway Enrichment Analysis (MPEA). (**A**) Traceability analysis of differential metabolites between CG and C-PAH. The horizontal axis represents the source category of metabolites; the vertical axis represents the number of metabolites. (**B**) Venn diagram displaying the number of metabolites in the lower airway of the host, microbiota, and co-metabolism. (**C**) MPEA based on host, microbial community, and metabolite subgroups in co-metabolism (hypergeometric test, log 0.05 (P) > 1). (**D**) Traceability analysis of 3 differential metabolites between the C-PAH and C-NPAH groups, with the horizontal axis representing the source category of metabolites and the vertical axis representing the number of metabolites. (**E**) Venn diagram showing the number of enriched metabolic pathways in MPEA based on co-metabolism sources. (**F**) Nicotinate and nicotinamide metabolism was enriched (hypergeometric test, log 0.05 (P) > 1). MNAM, N1-methylnicotinamide.

A total of 88 differential metabolites were identified between the CG and C-PAH group, among which 70 were derived from dietary sources (Figure 4A) and 22 were of both host and microbial origin (Figure 4B, Table S12). Pathway enrichment analysis revealed that host-derived metabolites were primarily enriched in glycerophospholipid metabolism and ether lipid metabolism (Table S13). In contrast, microbially derived metabolites were enriched in pathways related to cutin, suberine, and wax biosynthesis; pinene, camphor, and geraniol degradation; and the biosynthesis of phenylalanine, tyrosine, and tryptophan (Figure 4C, Table S14). Further comparison between the C-NPAH and C-PAH groups identified three differential metabolites—MNAM, 2-piperidone, and DL-2-aminooctanoic acid—all of which are potentially derived from food sources (Figure 4D, Table S15). Among them, MNAM is recognized as a co-metabolite, suggesting that it may originate from either the host or the gut microbiota (Figure 4E). Enrichment analysis indicated that MNAM is predominantly involved in the nicotinate and nicotinamide metabolism pathway (Figure 4C, Table S16).

### 3.8. The Correlation Between Bacteria and Metabolites and the Clinical Indicators in Each Group of Samples

**Figure 5:**
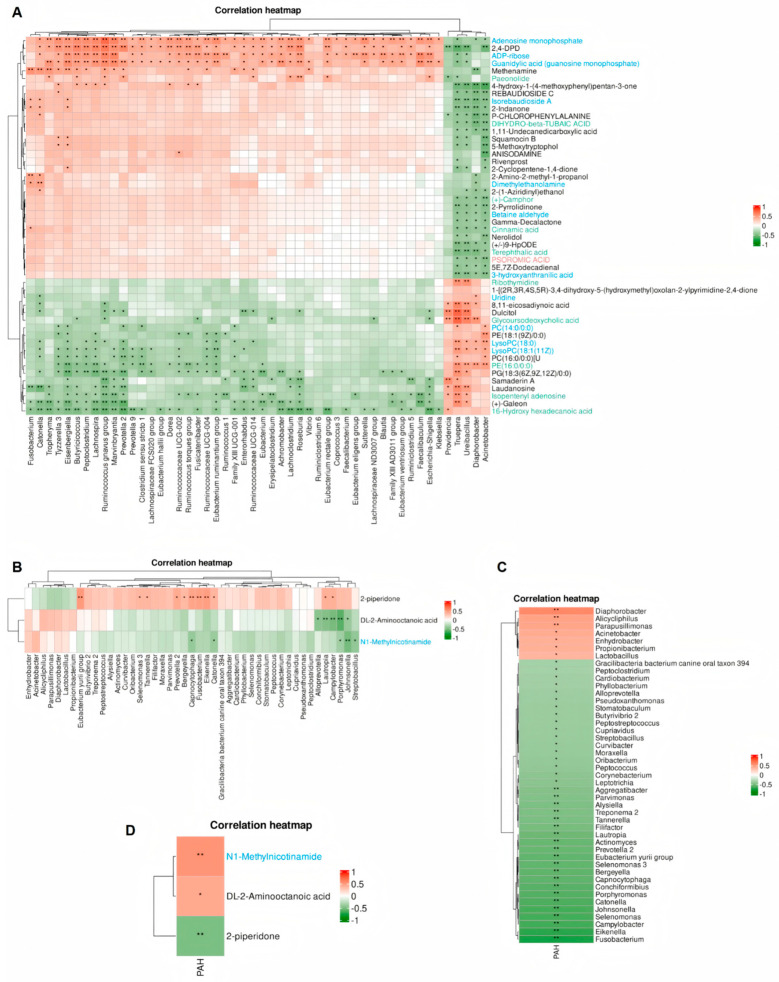
Correlation analysis between microbes at the genus and metabolite levels. (**A**) Association analysis of differential bacteria and metabolites between the CG and C-PAH group. (**B**) Association analysis of differential bacteria and metabolites between the C-PAH and C-NPAH groups. (**C**) Association analysis between differential bacteria of the C-PAH and C-NPAH groups and PAH. (**D**) Association analysis between differential metabolites of the C-PAH and C-NPAH groups and PAH. Correlations were analyzed by using Spearman’s correlation analysis. Red indicates positive correlation, and green indicates negative correlation. For pairwise group comparisons of individual microbial taxa or metabolites, the *t*-test was performed, with the Benjamini–Hochberg FDR correction applied for multiple testing (*p* < 0.05) * *p* < 0.05, ** *p* < 0.01. The above microorganisms are identified at the genus level. PAH, Pulmonary arterial hypertension.

In order to further investigate the differential bacterial and metabolite profiles, we first analyzed the interactions between the labeled differential genus-level microorganisms and differential metabolites between the CG and C-PAH group (Figure 5A, Table S17). We chose to study the genus-level characteristic microorganisms obtained from LEfSe analysis. As shown in Figure 5A, g_Alicycliphilis in the C-PAH group is positively correlated with Glycoursodeoxycholic acid (R = 0.55) but negatively correlated with Terephthalic acid (R = −0.38). g_Providencia is negatively correlated with Paeonide (R = −0.43) but positively correlated with Isopenyl Adenosine (R = 0.38) and Glycoursodeoxycholic acid (R = 0.48). In order to identify the relationship between differential bacteria and metabolites between the C-PAH and C-NPAH groups, we first analyzed and plotted a correlation heatmap. The results showed that g_Fusobacterium, g_Campylobacter, the g_Eubacterium yurii group, and g_Eikenella were positively correlated with 2-piperidone (*p* < 0.01), with R values of 0.48, 0.41, 0.52, and 0.50, respectively (Figure 5B, Table S18). At the same time, we also noticed that 45 species of microorganisms at the genus level correlated with the binary variable PAH for diagnosing diseases, 38 of which were negatively correlated and 7 of which were positively correlated (Figure 5C, Table S19). The correlation analysis between differential metabolites and clinical indicators showed a negative correlation between 2-piperidone and PAH (R = −0.51), while MNAM and DL-2-Aminoocatanoic acid were positively correlated with PAH, with R values of 0.55 and 0.44, respectively (Figure 5D, Table S20).

### 3.9. Microbiota-Associated Metabolites Link Lower Respiratory Tract Microbes to PAH Phenotypes

**Figure 6:**
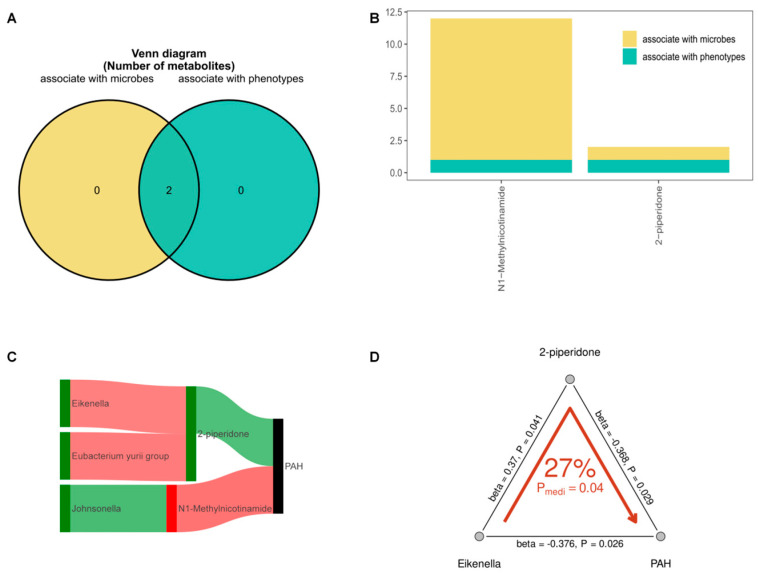
Mediation linkages between microbiota, metabolites, and clinical parameters. (**A**,**B**) The number of differential metabolites that are significantly associated with microbial species and phenotype parameters were identified and presented in a Venn diagram and bar plot. *p* values are calculated using the hypergeometric test. The Y-axis indicates the log 0.05 transformation of *p* values. Metabolic pathways with log 0.05 *p* values greater than 1 are considered statistically significant. (**C**) Metabolites that correlate with both the microbiome and PAH were further visualized in a Sankey network. The width of the flows in the Sankey network graph reflect the degree of correlation between bacteria and metabolites. The wider the band, the stronger the correlation. (**D**) The mediating effect of 2-piperidone in the relationship between g_Eikenella and PAH (β = −0.376). PAH, Pulmonary arterial hypertension.

To investigate the potential diagnostic role of lower respiratory tract microbes in PAH, we conducted an integrated analysis of microbes, metabolites, and clinical phenotypes. As shown in the Venn diagram (Figure 6A), two metabolites, MNAM and 2-piperidone, were found to be associated with both microbial taxa and PAH phenotype, suggesting their potential role as microbe–host co-modulators. The contribution of each metabolite to microbial and phenotypic associations was further quantified (Figure 6B). MNAM showed strong dual associations, whereas 2-piperidone exhibited a relatively weaker, but still notable, link to both microbial communities and phenotype. A Sankey diagram demonstrated the specific microbial contributors to these metabolites: Eikenella and the Eubacterium yurii group were linked to increased levels of 2-piperidone, while Johnsonella was associated with MNAM production (Figure 6C). To evaluate the potential mediating effect of metabolites in the microbe–PAH axis, a mediation analysis was conducted. Notably, 2-piperidone was found to mediate 27% of the association between Eikenella and PAH (*p* < 0.05), with significant effect sizes observed along all three paths of the mediation model (β = 0.372, *p* = 0.041 for Eikenella to 2-piperidone; β = 0.368, *p* = 0.029 for 2-piperidone to PAH; and β = −0.376, *p* = 0.026 for Eikenella to PAH) (Figure 6D, Tables S21 and S22). These findings suggest that 2-piperidone may serve as a functional intermediary linking microbial dysbiosis to PAH development.

## 4. Discussion

Although children with left-to-right shunt congenital heart disease have cardiac structural abnormalities, not all will develop PAH in the early stages of the disease. This phenomenon suggests that, in addition to the abnormal cardiac anatomy, other factors may play an important role in the occurrence and development of PAH. This study aimed to explore the potential role of the lung microenvironment in congenital heart disease-related PAH by analyzing the microbial community changes and differential metabolites in BALF samples from patients with C-PAH and C-NPAH and individuals in a CG. The results showed significant differences in lung microbial and metabolite profiles between C-PAH and C-NPAH patients, suggesting that dysbiosis of the lung microenvironment may play an important role in the pathophysiological process of C-PAH.

This study found a significant difference in the composition of lung microbial communities between C-PAH and C-NPAH patients, especially at the phylum, the proportion of Firmicutes/bacteroidetes (F/B) ratio in C-PAH patients was significantly higher than in C-NPAH patients and CG. An increase or decrease in the F/B ratio is considered a sign of dysbiosis [17]. The former is typically associated with obesity and metabolic disorders, which may be linked to enhanced caloric extraction from food, fat deposition, and adipogenesis, as well as impaired insulin sensitivity [18,19]. In contrast, the latter is associated with diseases such as Inflammatory Bowel Disease (IBD) and Alzheimer’s disease [20,21], possibly due to reduced production of short-chain fatty acids (SCFAs), especially butyrate, and lipopolysaccharide-induced inflammation mediated by the TLR4/NFκB pathway [22].

In addition, our study also revealed that patients with C-NPAH exhibited greater bacterial uniformity, consistent with previous results suggesting that the microbiota of healthy lungs was relatively uniform [23]. The lung microbiota plays an important role in maintaining microecological stability. A change in microbiota diversity may weaken the resistance of microbial communities to pathogens, thereby increasing inflammation and aggravating PAH. Therefore, analysis of lung microbial community composition can be used to monitor lung health [24,25].

Analysis of microbial genera further revealed differences between C-PAH and C-NPAH patients. g_Lactobacillus was enriched in C-PAH patients. In studies on gut microbiota, g_Lactobacillus has been reported to be associated with anti-inflammatory effects [26,27]. However, it is also believed that the abundance and diversity of lung flora will be altered during chronic inflammation; this could mean an increase in the abundance of g_Lactobacillus and a decrease in the abundance of g_Prevotella [28]. In addition, a systematic review and meta-analysis reported that the abundance of g_Lactobacillus was higher in the airway microbiota of patients with AECOPD compared with healthy controls [29]. This is consistent with our findings, since the occurrence and development of PAH are closely related to the inflammatory response [30,31,32]. In our study, LEfSe analysis demonstrated that the characteristic bacterial genera of C-NPAH patients included g_Fusobacterium and g_Porphyromonas. Therefore, the microbial flora structure of C-NPAH tends to be more similar to that of normal healthy people, despite their left-to-right shunt. In conclusion, this microbial community difference could regulate the host’s immune response and inflammatory state through various mechanisms. This may explain why some patients with congenital heart disease do not exhibit elevated pulmonary arterial pressure or develop it only at later disease stages.

Meanwhile, we noted that children with hereditary pulmonary arterial hypertension (HPAH), a key subtype of pediatric PAH, and patients with C-PAH share core pathophysiological changes: enhanced pulmonary vascular inflammation and vascular remodeling [33]. Given our key finding that microbiota dysbiosis in C-PAH is closely linked to inflammation, we hypothesize HPAH may also progress via a similar microbiota–inflammation–vascular remodeling axis. Key biomarkers identified herein, such as 2-piperidone, likely derive from gut microbiota metabolism, suggesting HPAH may involve a comparable gut-lung axis regulatory mechanism. However, pathogenic mutations (e.g., BMPR2, TBX4) in HPAH patients may indirectly alter airway microecology by regulating immune-inflammatory responses. Subtype-specific differences in microbiota composition and metabolite profiles between HPAH and C-PAH remain to be verified in targeted studies.

Currently, the role of MNAM in cardiovascular diseases remains unclear. In vivo, MNAM is produced from methylates nicotinamide (NAM) via nicotinamide N-methyltransferase (NNMT). Higher levels of MNAM have been reported in individuals with coronary artery disease. However, MNAM can also play a vascular-protective role by increasing endothelial prostacyclin (PGI2) and nitric oxide (NO) levels [34]. Fedorowicz et al. showed that plasma MNAM concentrations were elevated in patients with idiopathic pulmonary arterial hypertension (IPAH) and that PAH progression was associated with activation of the endogenous NNMT-MNAM pathway [35]. In our study, differential metabolite analysis of BALF samples from C-PAH and C-NPAH patients showed that MNAM was more highly expressed in the C-PAH group (Figure S4A, Table S26), which is consistent with the findings of Fedorowicz et al. As we collected BALF samples, our results reflect the characteristics of local metabolic changes.

Notably, due to the rarity of HPAH [36], data and reports on its associated micro-biota and metabolites remain scarce. Extending the above findings, the NNMT-MNAM pathway, consistently elevated in both IPAH and C-PAH, may also contribute to HPAH pathogenesis. Nevertheless, the specific local expression characteristics of this pathway in the airways of HPAH patients, its causal relationship with disease onset, and whether HPAH exhibits a microbiota-metabolite interaction mechanism similar to that of C-PAH still require targeted verification in subsequent studies by enrolling an adequate number of pediatric HPAH patients.

The mediation analysis results of this study further revealed the complex interaction between microbe, metabolite, and host in disease. *g_Eikenella* had a direct positive effect on PAH (β = 0.372, *p* = 0.041), while the indirect effect on PAH through the intermediary variable (2-piperidone) was also significant (β = −0.376, *p* = 0.026). This suggests that microbial community changes may influence the occurrence of PAH by affecting the levels of specific metabolites. *g_Eikenella* is a conditional pathogen that typically inhabits dental plaque. It has also been reported in the respiratory, digestive, and urinary tracts [37,38]. Many studies have shown that the enrichment of oral bacteria in the lower respiratory tract is a common phenomenon that has a complex impact on lung pathology [39,40]. This also verified that our results regarding *g_Eikenella* in the lung were reliable. Our study suggests 2-piperidone, as a new biomarker for the rapid and accurate diagnosis of diabetic retinopathy [41]. In addition, the concentration of 2-piperidone in urine correlates with the expression and activity of cytochrome P450 2E1 (CYP2E1); therefore, 2-piperidone is a potential indicator of CYP2E1 activity. Although our traceability analysis indicates that 2-piperidone is derived from food, the above research suggests that 2-piperidone can also exist in peripheral circulation. Zhou et al.’s study further elaborated that four intestinal bacterial strains can generate 2-piperidone from 5-amino valeric acid (5AVA) [42]. Therefore, our study also proves the influence of the gut–lung axis on the pathogenesis of PAH from another perspective [43,44,45].

Notably, this study focuses on a pediatric population (individuals under 14 years of age) whose pulmonary microenvironment differs markedly from that of adults. These differences may influence the patterns of microbiota–metabolite–host interactions. From a developmental standpoint, the pediatric lung is still undergoing maturation. The airway epithelial barrier function has not yet fully stabilized and mucosal immunity, such as alveolar macrophage phenotypes and cytokine secretion profiles, exhibits age-dependent characteristics. This immunologically immature state may render the pulmonary microbiota more susceptible to environmental perturbations [46]. For example, the increased F/B ratio observed in children with C-PAH in this study was significantly higher than that reported in adult PAH cohorts [47], suggesting that the degree of microbiota dysbiosis during childhood may be more closely associated with the progression rate of pulmonary vascular remodeling. In addition, as previously noted, the increased abundance of *g_Lactobacillus* in the C-PAH group may be related to the colonization preferences of specific strains during childhood. Unlike the well-documented anti-inflammatory effects of Lactobacillus in the adult gut [26,27], its enrichment in the pediatric lung may represent a form of compensatory colonization during the early establishment of mucosal immunity. The mechanisms by which Lactobacillus regulates local inflammation—particularly through metabolite production such as short-chain fatty acids—may differ at the strain level compared to adults. These strain-specific functional differences warrant further investigation, ideally through isolation and characterization of the dominant strains in pediatric populations.

In conclusion, this exploratory microbial metabolite–host–disease correlative model provides a preliminary perspective for understanding the potential mechanisms of PAH and may provide a theoretical basis for developing diagnostic and therapeutic strategies based on the microbiome and metabolome.

## 5. Limitations and Perspectives

This study has some limitations. First, it focused on the microbiome and metabolome but did not explore the host genome in depth. Therefore, future research can combine multi-omics methods to comprehensively analyze the complex relationships among microorganisms, metabolites, and host genes. In addition, the mediation analysis in this study is based only on correlation analysis, so causality cannot be determined. Therefore, future research can further verify the causal relationship between microbe, metabolite, host, and disease through experimental intervention or longitudinal cohort studies with larger sample sizes. Finally, the small sample size may limit our ability to effectively explain the errors caused by individual differences. Despite these limitations, this study provides new insights into the differences between the lung microbiome and metabolome in C-PAH and C-NPAH patients, as well as the interactions between these different omics layers.

## 6. Conclusions

Through integrative analysis of the microbiome and metabolome through BALF samples, this study identified, for the first time, pulmonary microbial dysbiosis and characteristic metabolites (such as 2-piperidone, MNAM) in patients with C-PAH. These exploratory findings have identified candidate microbial and metabolic biomarkers (e.g., 2-piperidone, MNAM) for the early noninvasive identification of irreversible pulmonary vascular remodeling. However, the practical utility of these biomarkers requires rigorous validation in large-scale independent cohorts. Meanwhile, this study lays a solid foundation for elucidating the pathological mechanisms of C-PAH and exploring potential therapeutic targets.

## Figures and Tables

**Table 1:** Inclusion and Exclusion Criteria of Participants.

Inclusion Criteria	C-PAH	C-NPAH	CG
Confirmed left-to-right shunt CHD (ASD/VSD/PDA or combined malformations); PASP > 35 mmHg (transthoracic ultrasound); PVR > 3 Wood units (right heart catheterization); Age < 14 years; Written informed consent from guardians.	Applicable		
Confirmed left-to-right shunt CHD (same types as C-PAH); PASP ≤ 35 mmHg (transthoracic ultrasound); Age < 14 years; Written informed consent from guardians		Applicable	
No respiratory symptoms in 4 weeks prior to enrollment; Normal chest X-ray; No known pulmonary/CHD/systemic diseases; Age < 14 years; Written informed consent from guardians.			Applicable
**Exclusion Criteria (Applicable to all groups)**			
Right-to-left shunt CHD or congenital pulmonary vascular malformation; Prior thoracic/heart surgery (C-PAH/C-NPAH: prior to CHD diagnosis); C-PAH/C-NPAH: combined left heart failure or primary left heart valve disease; Oral/nasal antibiotic use within 4 weeks prior to enrollment; Premature birth (gestational age < 37 weeks) or in vitro fertilization; History of immunosuppressive drug use; Oral/respiratory infection within 4 weeks prior to enrollment; Known PAH-related diseases (autoimmune/liver/kidney/hematological diseases).			

CHD, congenital heart disease; PAH, pulmonary arterial hypertension; C-PAH, CHD with PAH; C-NPAH, CHD without PAH; CG, healthy control group, ASD, atrial septal defect; VSD, ventricle septal defect; PDA, patent ductus arteriosus; PASP, pulmonary artery systolic pressure.

**Table 2:** Personal and echo parameters of participants.

Descriptive Measurements	C-PAH (n = 15)	C-NPAH (n = 16)	CG (n = 16)	Z	*p* Value
Sex, male (%)	8 (53.3%)	8 (50.0%)	12 (75.0%)	0.1631	
Age, y; median (IQR)	0.83 (0.4–2.5)	3.0 (2.0–3.8)	4.0 (3.5–7.2)		0.016
Weight, Kg; median (IQR)	8.20 (6.3–11.5)	14.50 (9.7–15.5)			0.100
Diagnosis					
ASD	2(10.5%)	7 (28.0%)		0.4098	0.682
VSD	7 (36.8%)	10 (40%)		0.595	0.5518
ASD + PS	0	1 (4.0%)		0.9843	0.325
ASD + VSD	8 (42.1%)	6 (24.0%)		2.213	0.0269
VSD + PDA	1 (5.3%)	0		1.05	0.2938
ASD + VSD + PDA	1 (5.3%)	1 (4.0%)		1.05	0.2938
Echo					
LPA, mm	7.47 ± 1.93	6.60 ± 1.29			0.079
RPA, mm	7.68 ± 2.08	6.68 ± 1.60			0.077
MPAD, mm	16.05 ± 4.39	13.80 ± 2.87			0.046
Ao, mm	11.11 ± 3.51	13.04 ± 2.81			0.054
LVEDD, mm	31.37 ± 7.20	29.48 ± 4.97			0.321
AVPV, m/s	1.06 ± 0.13	1.12 ± 0.33			0.444
PVSV, m/s	1.36 ± 0.34	1.11 ± 0.56			0.093
EF, %	67.74 ± 5.39	68.84 ± 3.01			0.393
RV (long axis, mm)	15.32 ± 4.19	14.64 ± 3.74			0.576
LA (long axis, mm)	20.53 ± 6.38	19.44 ± 3.82			0.486
PASP, mmHg	61.58 ± 16.38				
Treatment					
CPB open surgery	19 (100%)	25 (100%)			

Values are median (interquartile range), n (%), or mean ± SD. Data were compared using *t*-tests. IQR, interquartile range; CHD, congenital heart disease; PAH, pulmonary arterial hypertension; C-PAH, CHD with PAH; C-NPAH, CHD without PAH; CG, healthy control group; ASD, atrial septal defect; VSD, ventricle septal defect; PS, pulmonary stenosis; PDA, patent ductus arteriosus; LPA, left pulmonary artery; RPA, right pulmonary artery; MPAD, main pulmonary artery diameter; Ao, Aorta; LVEDD, left ventricular end-diastolic diameter; AVPV, aortic valve peak velocity; PVSP, pulmonary valve flow velocities; EF, ejection fraction; RV, right ventricular; LA, left atrium; PASP, pulmonary artery systolic pressure; CPB, cardiopulmonary bypass.

## Data Availability

The data presented in the study are deposited in the China National Center for Bioinformation (https://www.cncb.ac.cn/?lang=en) repository, accession number OMIX001230. The data supporting this study’s findings are also available upon reasonable request from the corresponding authors.

## Supplementary Materials

The following supporting information can be downloaded at: https://www.mdpi.com/article/10.3390/jcdd13010032/s1, Figure S1: Flattened data comprehensively captures the diversity of the samples. (A) Rarefaction curves of three samples. The horizontal axis of the curve represents the number of sequencing reads (label = 0.03); the vertical axis represents the observed OTUs. (B) Shannon–Wiener Curves of three samples. The horizontal axis (Number of Reads Sampled) represents the number of sequencing reads, i.e., the sequencing depth (label = 0.03). The vertical axis (Shannon Index) represents the Shannon–Wiener index, i.e., the diversity of the sample. (C) Relative abundance of three groups of microbial samples at the phylum level. (D) Relative abundance of three groups of microbial samples at the genus level; Figure S2: Results of LEfSe with cladogram and bar plots from the phylum to genus levels. (A) Each node in the graph represents a specific microbial OTU from phylum to genus. The sizes and colors of the nodes indicate the relative abundance and statistical significance of this OTU in different groups. The branches in the figure connect different levels of OTUs, from wider taxa (such as phyla) to more specific taxa (such as genera). (B) Each bar in the graph represents a specific microbial taxon, from phylum to species. The color of the bars indicates different sample groupings. The x-axis in the figure represents LDA > 3; Figure S3: PLSDA validation results. (A), (b), and (c) show the validation results for CG vs. C-PAH, CG vs. C-NPAH, and C-PAH vs. C-NPAH, respectively. The abscissa represents correlation. The values range from −1 to 1, where 1 indicates a complete positive correlation, −1 indicates a complete negative correlation, and 0 indicates no linear correlation. The ordinate represents the intercept of R^2^ or Q^2^; Figure S4: Comparison of differential metabolite expression levels between groups. Box plots (A), (B), and (C) show the differential metabolites N1-methylnicotinamide, 2-piperidone, and DL-2-aminooctanoic acid. *p* < 0.05 was considered statistically significant. * *p* < 0.05, ** *p* < 0.01, *** *p* < 0.001; Table S1: Top 20 microorganisms at the phylum level and F/B ratios in the 3 groups; Table S2: Top 20 microorganisms at the phylum level; Table S3: Taxonomic of Microorganisms; Table S4: Shannon, Richness, and Pielou for each group; Table S5: Indicators for 3 groups; Table S6: LEfSe analysis on the genus of the 3 sample groups; Table S7: Principal Component Analysis of Metabolic; Table S8: PLSDA validation; Table S9: Metabolite Difference Analysis (Showed 88 metabolites between CG and C-PAH); Table S10: Metabolite Difference Analysis (Showed 3 metabolites between C-NPAH and C-PAH); Table S11: Venn C-NPAH vs. C-PAH and C-PAH vs. CG; Table S12: Metabolite traceability analysis; Table S13: Metabolic Pathway Enrichment Analysis (Host); Table S14: Metabolic Pathway Enrichment Analysis (Microbiota); Table S15: C-PAH vs. C-NPAH Metabolites Origin (3 differential metabolites); Table S16: Results of MPEA and Co-Metabolism (C-PAH vs. C-NPAH); Table S17: Interactions Between the Genus-level Microorganisms and Differential Metabolites Between CG and C-PAH (R Values); Table S18: Interactions Between the Genus-level Microorganisms and Differential Metabolites Between C-NPAH and C-PAH (P Values); Table S19: Microorganisms at the Genus Level Correlated with the Binary Variable PAH for Diagnosing; Table S20: Metabolites Correlated with the Binary Variable PAH for Diagnosing; Table S21: Mediation Model Params; Table S22: Mediation Model Summary; Table S23: Mobile Phase Gradient Elution; Table S24: Mass Spectrometry Parameters; Table S25: Equipment; Table S26: Expression of differential metabolite; Table S27: DESeq2(all); Table S28: ALDEx2(all).

## Abbreviations

The following abbreviations are used in this manuscript:

CHD	Congenital heart disease
PAH	Pulmonary arterial hypertension
IPAH	Idiopathic pulmonary arterial hypertension
BALF	Bronchoalveolar lavage
RF	Random forest
MNAM	N1-methylnicotinamide
NAM	Methylates nicotinamide
NNMT	Nicotinamide N-methyltransferase
LTRS-CHD	Left-to-right shunt-CHD
PASP	Pulmonary artery systolic pressure
OTU	Operational taxonomic unit
QC	Quality control
PVR	Pulmonary vascular resistance
MPAD	Main pulmonary artery diameter
PCA	Principal component analysis
F/B	Firmicutes/bacteroidetes
IBD	Inflammatory bowel disease
SCFAs	Short-chain fatty acids
CYP2E1	Cytochrome P450 2E1
5AVA	5-amino valeric acid
HPAH	Hereditary pulmonary arterial hypertension
