# Supplementary figures and images for: Microbiome–Metabolome Axis in BALF Reveals Novel Diagnostic Biomarkers for Congenital Heart Disease-Associated Pulmonary Arterial Hypertension
Source: J Cardiovasc Dev Dis. 2026 Jan 6;13(1):32. doi: 10.3390/jcdd13010032 (PMC12842009; doi:10.3390/jcdd13010032)

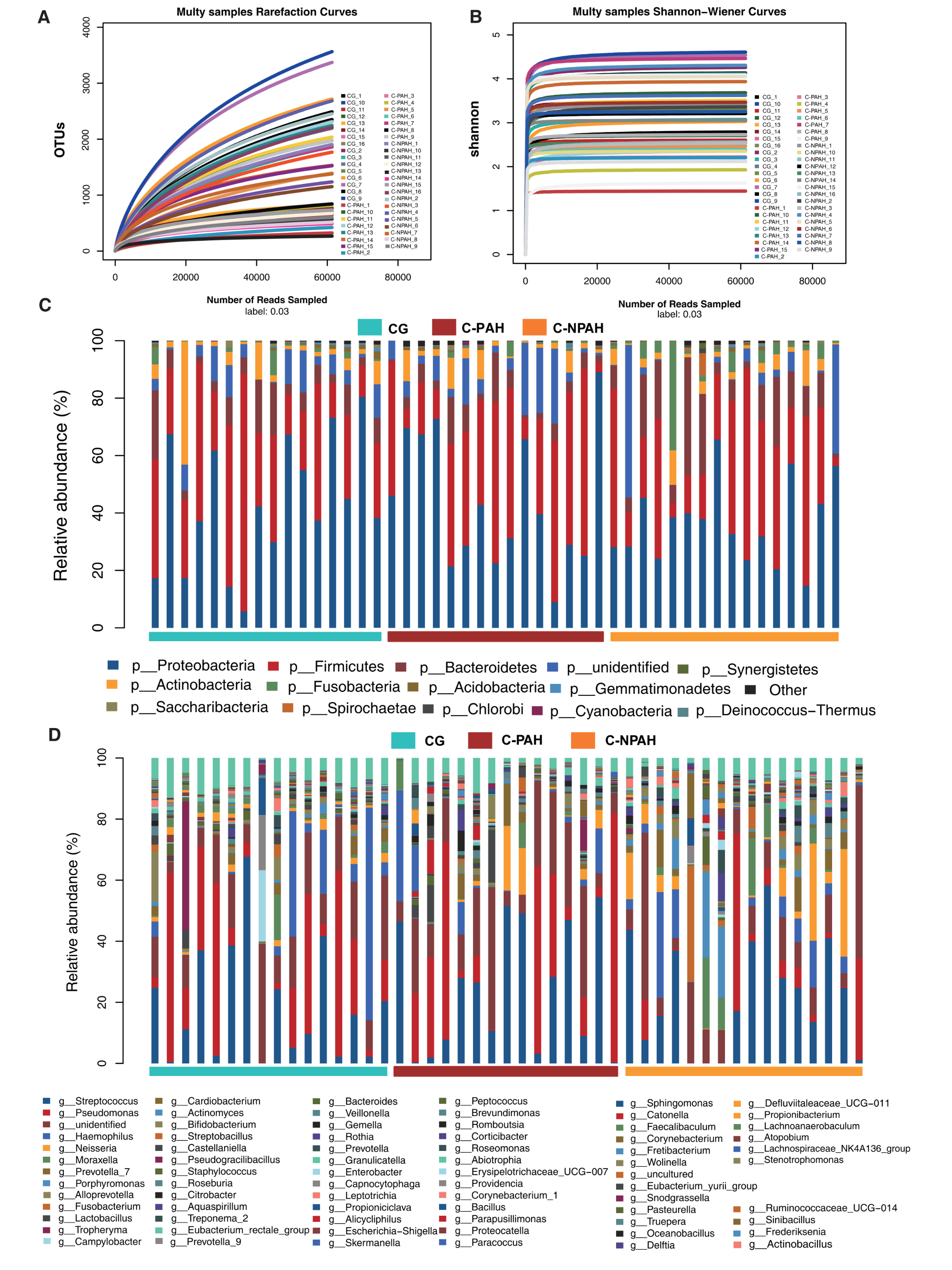

Supplement: Supplementary file 1 [file jcdd-13-00032-s001.zip › Figure S1..tif]

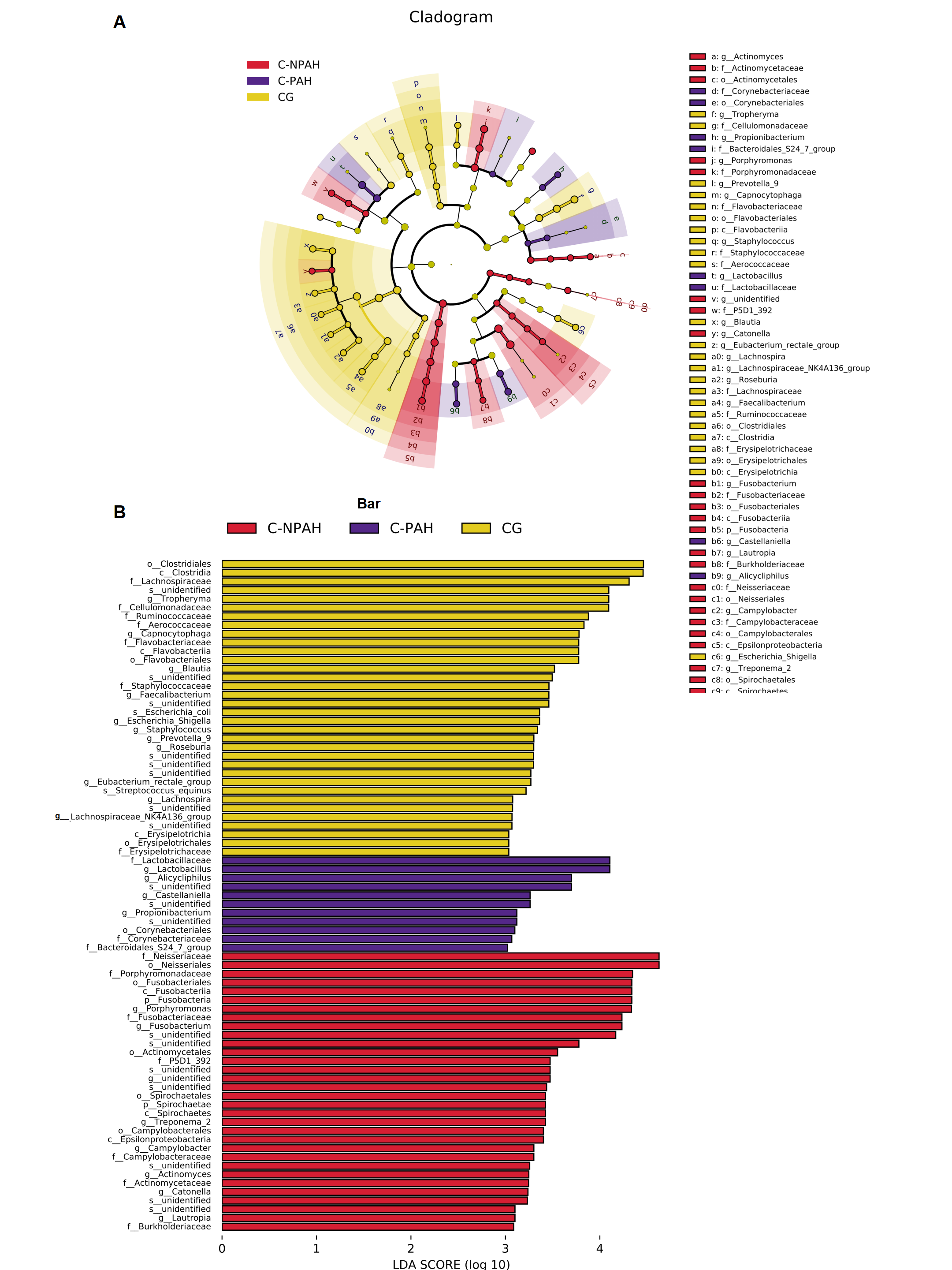

Supplement: Supplementary file 1 [file jcdd-13-00032-s001.zip › Figure S2..tif]

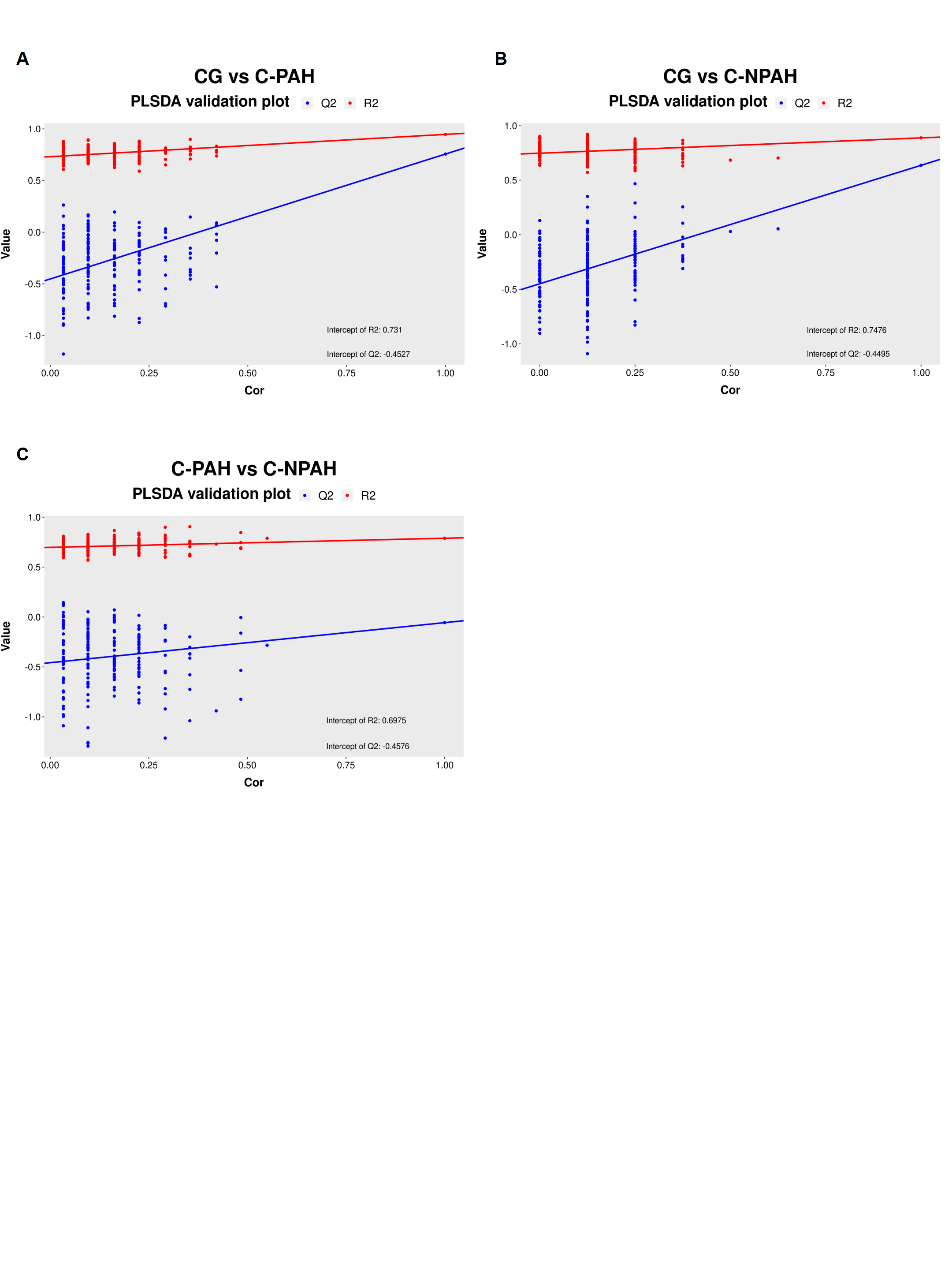

Supplement: Supplementary file 1 [file jcdd-13-00032-s001.zip › Figure S3..tif]

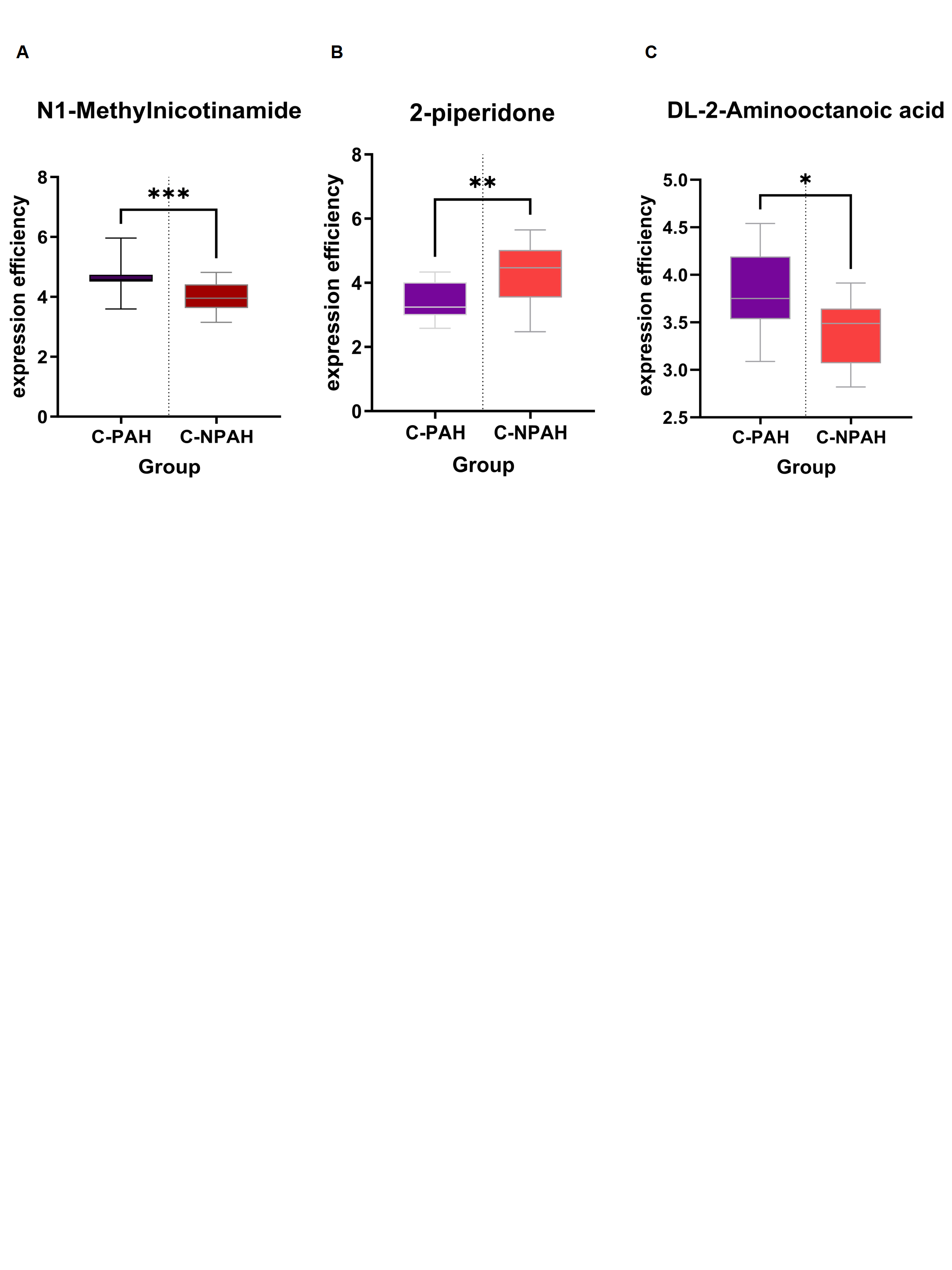

Supplement: Supplementary file 1 [file jcdd-13-00032-s001.zip › Figure S4..tif]
